# Simulation of three dimensional diffraction patterns as aid of structural analysis for complex epitaxial films

**DOI:** 10.1038/s41598-025-01310-w

**Published:** 2025-05-26

**Authors:** Yajun Tao, Erhao Peng, Qingyu He, Xinyan Chen, Jiangxiao Li, Yuting Wang, Yongqi Dong, Zhe Sun, Zhenlin Luo

**Affiliations:** National Synchrotron Radiation Laboratory, University of Science and Technology of China, Hefei, 230026 China

**Keywords:** Epitaxial film, Synchrotron three-dimensional diffraction, 3D-RSM, Surface diffraction, Simulation, Applied physics, Materials science

## Abstract

Thin film is the form of material that most closely resembles the silicon-based integrated circuits (IC) and therefore has attracted tremendous attention over the past decades due to its potential applications in integrating functional devices on IC chips. The structural characterization of thin films, especially epitaxial film with complex structure has been a long-term challenge until the emergence of synchrotron three-dimensional diffraction technique (3D-RSM). 3D-RSM is a technique that can effectively collect various structural information of epitaxial films, such as crystal lattice, strain, domain variants, and oxygen octahedral rotation. Now, interpreting the massive experimental data of 3D-RSM becomes the biggest obstacle that is confronted by the researchers. In this work, we proposed a strategy that utilizes simulated 3D-RSM diffraction patterns as aid of data analysis. With this approach, the one-to-one correspondence between diffraction spots and domain variants, as well as the quantitative lattice constants and crystal system have been identified in sequence for two typical cases, either epitaxial PbTiO_3_/SmScO_3_(001) film or (CoCrFeMnNi)_3_O_4_ alloy film epitaxially grown on LaAlO_3_(001) substrate. Further, systematic simulations of 3D-RSM patterns for epitaxial films belonging to every of the seven crystalline symmetries were performed and exhibited, assuming the films are grown on a (001)-oriented cubic substrate. This work sheds light on more effective data analysis of 3D-RSM, i.e., more effective structural characterization of complex epitaxial films.

## Introduction

Epitaxy growth possesses huge potential in integrating functional devices, and has been widely applied in applications such as high-density information storage^1–4^, superconductivity^5,6^, and flexible devices^7–9^. The physical properties of these epitaxial films are majorly dominated by their structure^10^, but characterizing this structure is a long-term challenge^11^, particularly for complex epitaxial films^12^, such as epitaxial composite films^13^, multi-domain films^14,15^ or films with oxygen octahedral rotation^16^.

Recently, characterization techniques such as synchrotron three-dimensional diffraction (also named as three-dimensional reciprocal space mapping, 3D-RSM) have been developed, leveraging the high photon flux of synchrotron radiation and advanced X-ray detectors^17–19^. This 3D-RSM technique provides an excellent opportunity for structural analysis of complex epitaxial films^20–28^. However, data analysis of 3D-RSM remains challenging. The reason is that each domain in the epitaxial film behaves like a single crystal when diffracted by X-rays, resulting in a three-dimensional periodic lattice in the diffractive reciprocal space. Theoretically speaking, by measuring this reciprocal lattice, structural information such as lattice parameters and strain in the real space lattice can be obtained^23^. Nevertheless, an epitaxial film often possesses one or more crystalline phases and each phase has domain variants with different orientations of lattice, which result in multiple sets of diffraction spots in the reciprocal space and consequently cause serious challenges for interpretation.

A considerable strategy for 3D-RSM data analysis is simulation. Actually, several studies have been reported on simulating diffraction patterns of epitaxial films. For multi-phase epitaxial systems, qualitative simulation results have been provided for the 00*l* diffraction spots in the *hl* plane^29^. In the case of multi-domain systems, diagrams of reciprocal lattice points corresponding to possible domain variants have been presented^30^. However, these studies only simulated and exhibited two-dimensional diffraction patterns, simulation of three-dimensional diffraction patterns of epitaxial films has rarely been reported.

In this work, using simulated 3D-RSM patterns as aid, we not only recognized the origin of each diffraction spot but also provided quantitative structural information for two epitaxial film cases: PbTiO_3_/SmScO_3_ (001) and (CoCrFeMnNi)_3_O_4_ /LaAlO_3_(001). Furthermore, systematic simulations of 3D-RSM patterns for epitaxial films belonging to each of the seven crystalline symmetries were performed and exhibited, assuming the films are epitaxially grown on (001)-oriented cubic substrates.

## Results and discussion

### PbTiO_3_/SmScO_3_(001)

**Fig. 1:**
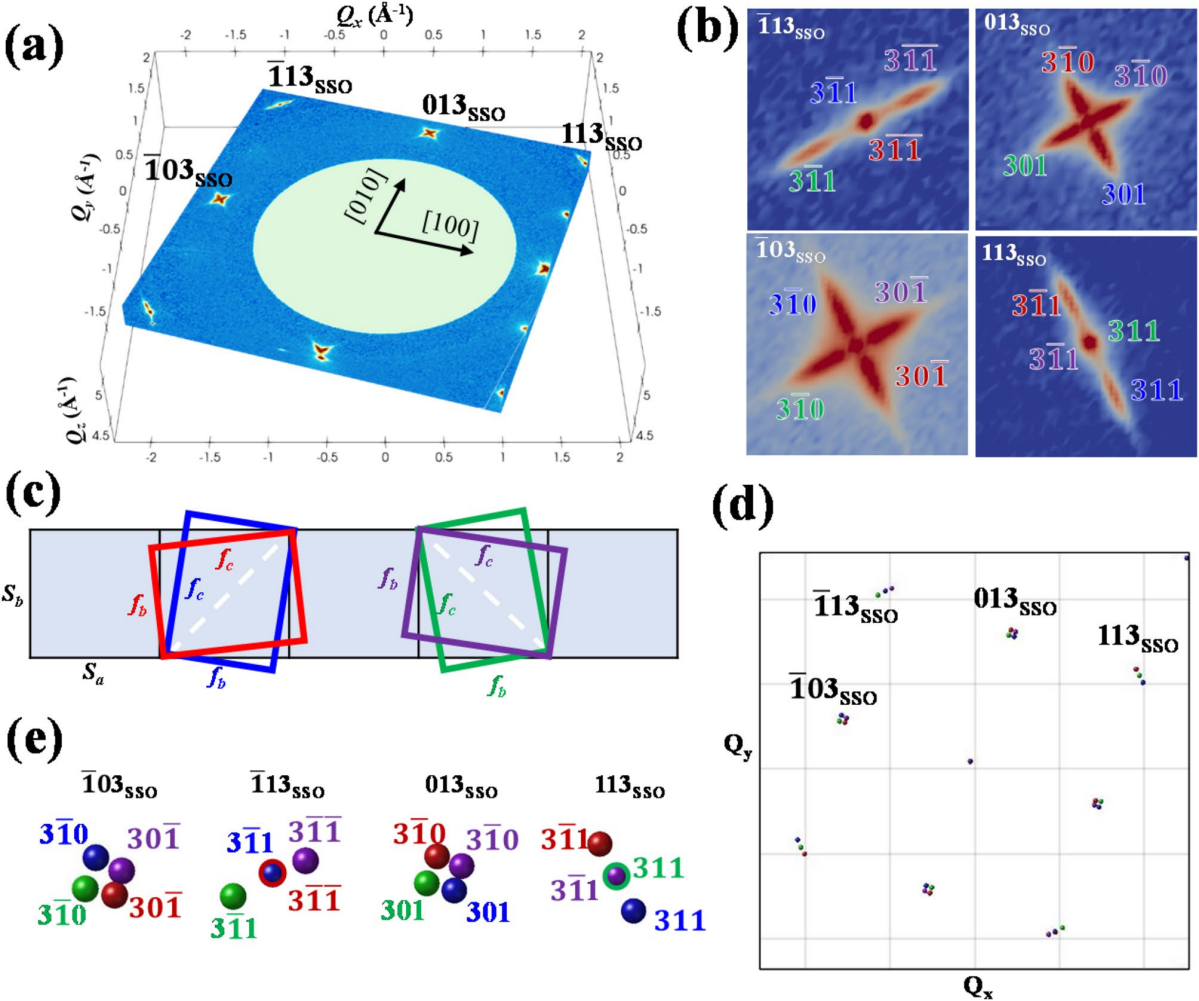
For the PbTiO_3_/SmScO_3_(001) epitaxial film, (**a**) experimental 3D-RSM result and (**b**) slice at *Qz* = 4.75 Å^−1^ show the PTO *3hk* diffraction spots. (**c**) Schematic epitaxy relationship of PTO film (100) plane on SSO substrate pseudo-cubic (001) plane and the corresponding (**d**) simulated 3D diffraction pattern for PTO and (**e**) its details. Please note that, the color indexed spots in (**b**, **e**) correspond to the same color PTO lattice illustrated in (c).

In the case of PbTiO_3_/SmScO_3_(001), ferroelectric PbTiO_3_ (PTO) film was first epitaxially grown on (001)-oriented pseudo-cubic SmScO_3_ (SSO) substrate and then patterned in dots with one micron diameter. The experimentally obtained 3D-RSM are presented in Fig. 1. In Fig. 1a, multiple selected 2D slices (contour maps) are presented to exhibit the diffraction intensity distribution in the 3D reciprocal space. The numbers with SSO subscript indicate the axis direction and diffraction index of SSO substrate. Above it, some special diffraction patterns appears and could be ascribed to PTO. Enlarged views of these patterns are shown clear in Fig. 1b, where the origins of each diffraction spot for PTO are recognized and indexed in colored numbers, with the colors corresponding to the diffraction spots of the thin film and their respective lattices. Actually, identification of these spots was the most difficult process, and was completed with the aid of simulation. According the above experimental data, we hypothesis that two types of PTO twin-domain are formed, with either [011] or $0{-}{\overline{\text{1}}}{-}{1}$ axis of PTO lattice aligns along/matches with the pseudo-cubic SSO lattice, as illustrated in Fig. 1c. The $3{-}{\overline{\text{1}}}{-}{1}$ and 301 diffraction peaks of PTO appear within the ${\overline{\text{1}}}1{3}$ and 013 Bragg spots of SSO, which are associated with the formation of the tetragonal a-domain of this PTO^31^. The simulated 3D-RSM patterns exhibited in Fig. 1d is well consistent with the experimental data. With the detailed simulated patterns in Fig. 1e, the origin of each experimental diffraction spot is identified in Fig. 1b. Subsequently, the lattice constants of PTO film are calculated according to Eq. (1) and the method is described in the methods part. The averaged PTO lattice parameters were measured to be *a*=*b*=3.9(3) Å, *c*=4.0(5) Å, *α* =*β* = *γ* =90°.

### (CoCrFeMnNi)_3_O_4_/LaAlO_3_(001)

**Fig. 2:**
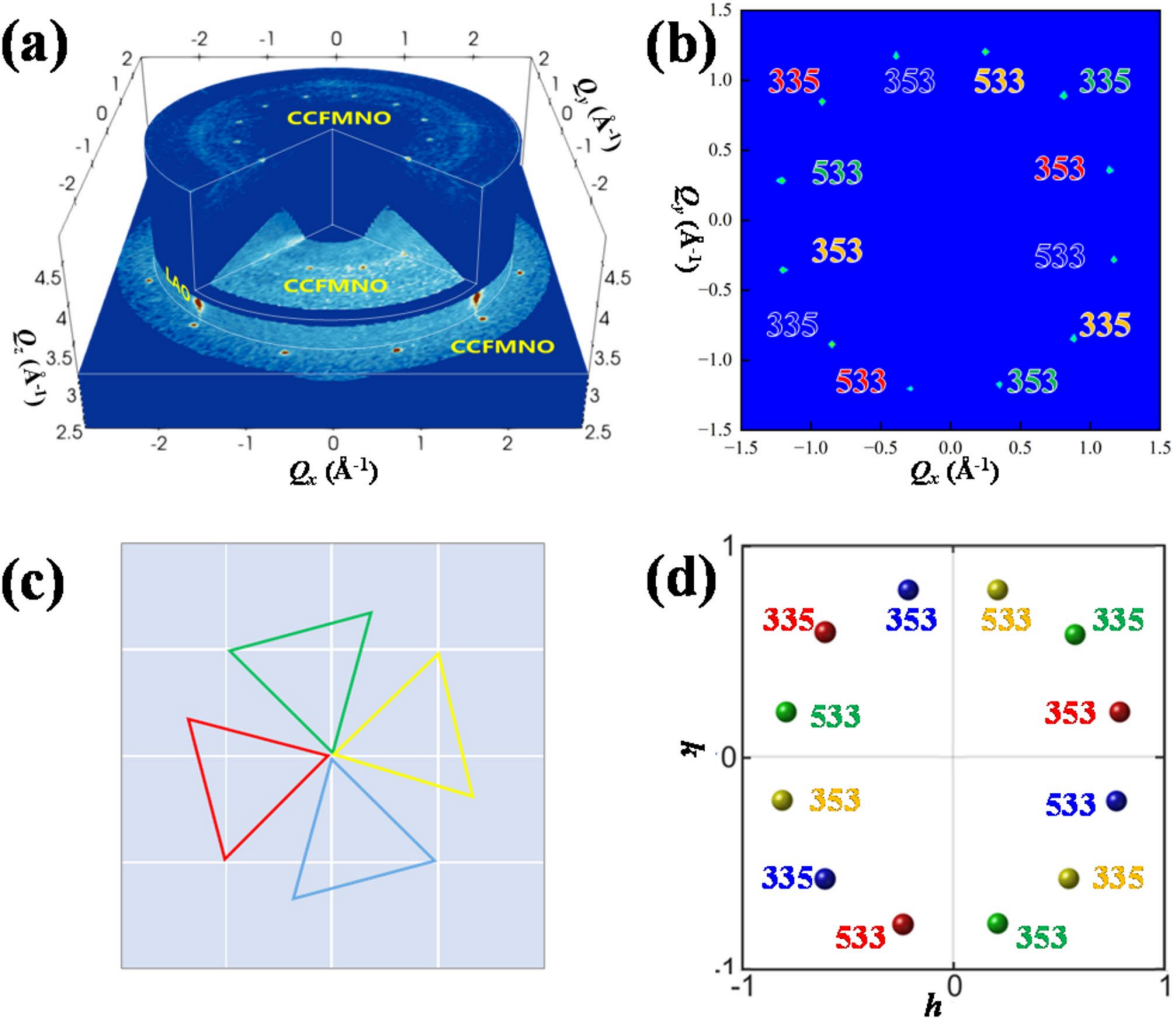
For the (CoCrFeMnNi)_3_O_4_/LaAlO_3_(001) epitaxial film, (**a**) experimental 3D-RSM result and (b) *QxQy* slice at *Qz* = 4.8 Å^−1^, (**b**) schematic epitaxy relationship of threefold symmetric CCFMNO (111) plane (colored) on fourfold symmetric (001) plane (white lattice) of pseudo-cubic LAO. (**d**) The simulated diffraction pattern of CCFMNO presented in LAO r.l.u. Please note that, the color indexed spots in (**b**, **d**) correspond to the same color CCFMNO lattice illustrated in (**c**).

In another case of (CoCrFeMnNi)_3_O_4_/LaAlO_3_(001), where magnetic multi-element alloy (CoCrFeMnNi)_3_O_4_ (CCFMNO) film was grown on (001)-oriented pseudo-cubic LaAlO_3_ (LAO) substrate, intriguing 12-fold diffraction patterns were found in the experiment, as shown in Fig. 2a,b. Simulation reveals the appearance of a specific epitaxy relationship, as illustrated in Fig. 2c, in which the film (111) crystal planes are parallel to the substrate (001) plane while one of the triangle edges in film lattice aligns along/ matches with the in-plane diagonal of substrate lattice. Since the film lattice has threefold symmetry and substrate lattice has fourfold symmetry, the resulting diffraction pattern shows 12-symmetry. As exhibited in Fig. 2b,d, the simulated 3D-RSM is in good agreement with the experimental data. With the recognized index of each diffraction spot as listed in Fig. 2b, the lattice constants of each CCFMNO domain variant were measured and the average value are *a* = *b* = *c* = 8.3(4)Å, *α* = *β* = *γ* = 90°, with an error less than 1%. This result verifies the CCFMNO film is cubic.

**Fig. 3:**
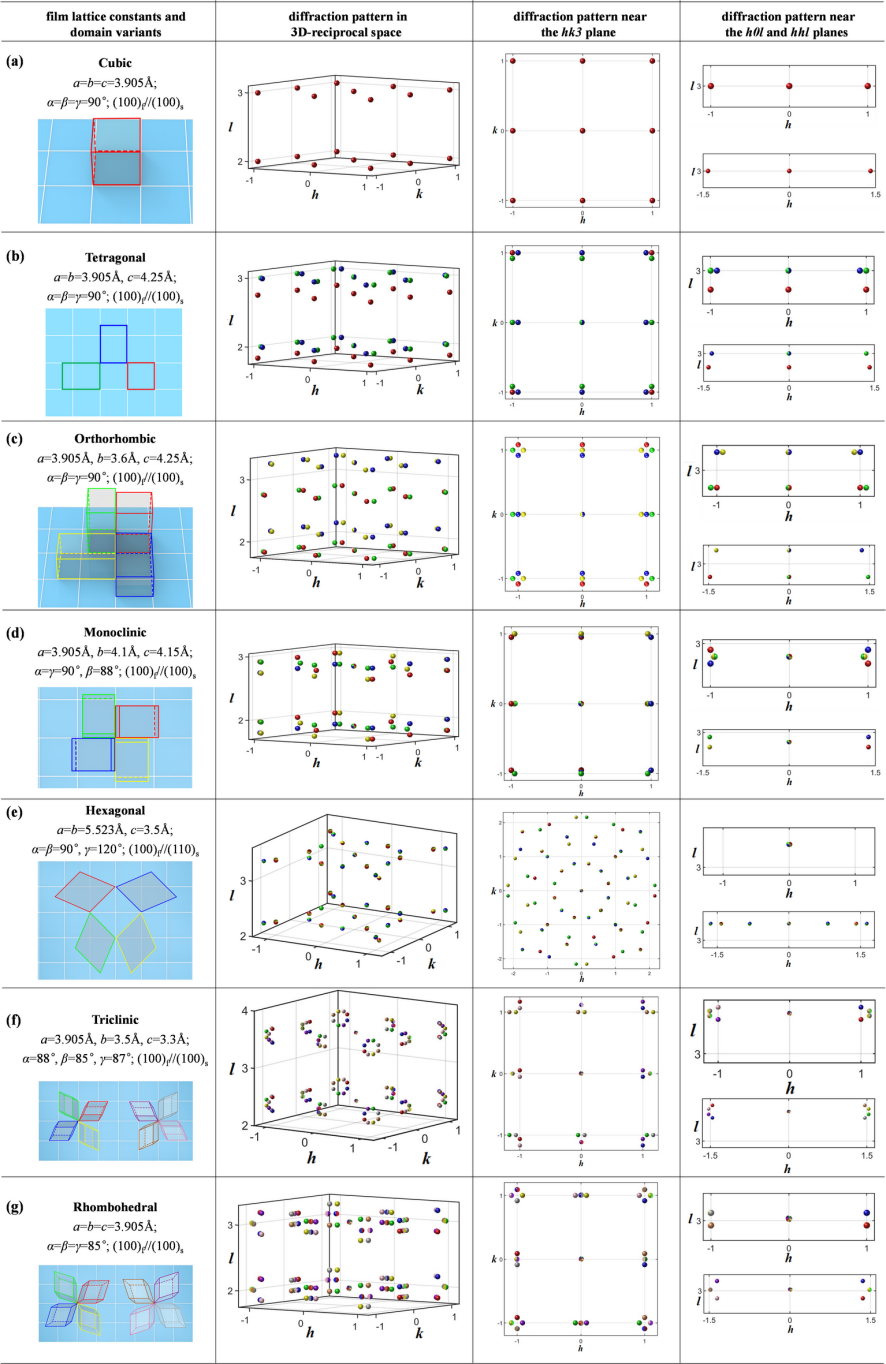
Simulated 3D diffraction patterns for films belonging to different crystalline symmetries: (**a**) cubic, (**b**) tetragonal, (**c**) orthorhombic, (**d**) monoclinic, (**e**) hexagonal, (**f**) triclinic and (**g**) rhombohedral. The assumed lattice constants and epitaxial relationship are listed in the 1st column, along with the simulated full reciprocal space diffraction patterns (2nd column), the specific patterns near the *hk*3-layer (3rd column),* h*0*l*-layer (4th column up) and *hhl*-layer (4th column down) presented in the r.l.u. of SrTiO_3_ substrate. SrTiO_3_ Bragg spots are not showed for abbreviation.

The above results show that simulation can be really helpful to 3D-RSM data analysis. Therefore, one could not stop looking forward to a systematic simulation performed before practical experiments. In the following, we perform a systematic simulation on the 3D-RSM patterns of films with all seven crystalline symmetries, i.e., cubic, tetragonal, orthorhombic, hexagonal, rhombohedral, monoclinic and triclinic. The technical details are described in the Methods part. Here, the substrate is assumed to be (001)-SrTiO_3_, while the film is assumed to be single phase but multi-domain variants, with lattice parameters and preset epitaxial relationship as listed in the first column of Fig. 3. Figure 3 provides a visual representation of the epitaxial growth of film with various crystalline symmetries on a cubic STO substrate, showcasing the unit cell schematics, simulated 3D diffraction pattern and the corresponding projections of simulated reciprocal lattice points on the *hk*3-, *h*0*l*-, and *hhl*-layers. For the given lattice constant and epitaxial relationship, the results show the different diffraction patterns for each case. From these simulating results, it could be concluded that:

Cubic Films: When grown epitaxial on a (001)-oriented cubic substrate, cubic films align perfectly with the substrate, resulting in a single-domain variant. This alignment is evident in the diffraction patterns, where the film’s diffraction spots coincide with those of the substrate, indicating a uniform and consistent crystalline structure.

Tetragonal Films: These films exhibit a more complex diffraction pattern due to the presence of three possible domain variants. The *c*-axis of the unit cell in these variants can be aligned along different directions, leading to a quadruple symmetry in the *hk*3-layer projection. The differences in the *a*/*c* lattice constants cause the diffraction spots to split along the *z*-direction in the *h*0*l* and *hhl* views, providing a clear distinction between the domains.

Orthorhombic Films: With the decrease in symmetry from tetragonal to orthorhombic, the films show a slightly different 3D-RSM. Assuming one lattice vector matches the substrate while the other two are significantly different, and this results in four domain variants. The *hk*3-view reveals diffraction points around the substrate’s Bragg spots, and the four domain variants showing distinct separations, according to the differences in lattice parameters.

Monoclinic Films: Even though monoclinic crystals can exhibit complex domain structures, the epitaxial monoclinic films considered here have only four domain variants when the *c*-axis is facing outward. The simulated patterns in the *h*0*l*-view are particularly distinctive, allowing for easy identification based on the presence or absence of certain diffraction spots.

Hexagonal Films: The epitaxy of hexagonal lattices on (001)-oriented cubic substrates results in four hexagonal domain variants. The *hk* view of the simulated diffraction pattern shows a clear 12-fold symmetry, which is a combination of the hexagonal lattice’s 6-fold symmetry and the cubic lattice’s 4-fold symmetry. This pattern is a key feature that distinguishes hexagonal films.

Triclinic Films: These films, with the lowest symmetry, exhibit eight sets of domain variants due to the *c*-axis tilting along both the *x* and *y* directions. The in-plane diffracted spots populate the Bragg region separately, presenting a quadruple symmetry relationship in the *hk*3-layer projection.

Rhombohedral Films: As a special case of triclinic symmetry, rhombohedral films have equal out-of-plane and in-plane lattice constants. This results in a quadruple symmetric diffraction pattern within the *hk*3-layer. However, the deviation of the crystal axes from 90° causes the 113 Bragg spot to split in the *hhl*-view, adding complexity to the pattern.

The above result show that our simulation study has successfully delineated the three-dimensional diffraction patterns for epitaxial films exhibiting all seven crystalline symmetries on a (001)-oriented cubic substrate. We believe these simulation outcomes will provide a valuable resource for the scientific community to accelerate 3D-RSM studies of complex epitaxial films. For example, by presenting the diffraction spot distributions in a broader reciprocal space, this work could aid researchers in developing optimized strategies for experimental data collection, thereby enhance the efficiency of utilizing synchrotron beam time—a precious but often limited resource. Moreover, the detailed and distinct patterns, as revealed in our simulations around 103 or 113, could serve as a quick reference for identifying the crystalline symmetry of the films, by comparing experimental data with the simulation results. Furthermore, under the guidance of this simulation, one is able to know with the index of each diffraction spots in the experimental collected diffraction pattern and thus to quantitatively calculated the crystalline lattice constants, as exemplified in the above Fig. 1 and Fig. 2. Please note that, the experimental pattern presented in Fig. 1e is not the same as the simulated tetragonal pattern in Fig. 3b. This discrepancy arise from the different epitaxial relationships and highlights the need for a more comprehensive simulation work.

As we advance in the field of epitaxial film research, the approach demonstrated in this study can be adapted for films grown with other epitaxial relationships or on other substrate orientations, potentially leading to the creation of a comprehensive 3D diffraction pattern database. This resource would be an invaluable asset in guiding future synchrotron radiation 3D diffraction experiments and their subsequent data analysis.

In summary, our simulations not only contribute to a better understanding of epitaxial film structures but also serve as a practical tool for researchers engaged in the study and application of these materials.

## Methods

### Simulation: basic concept and model

**Fig. 4:**
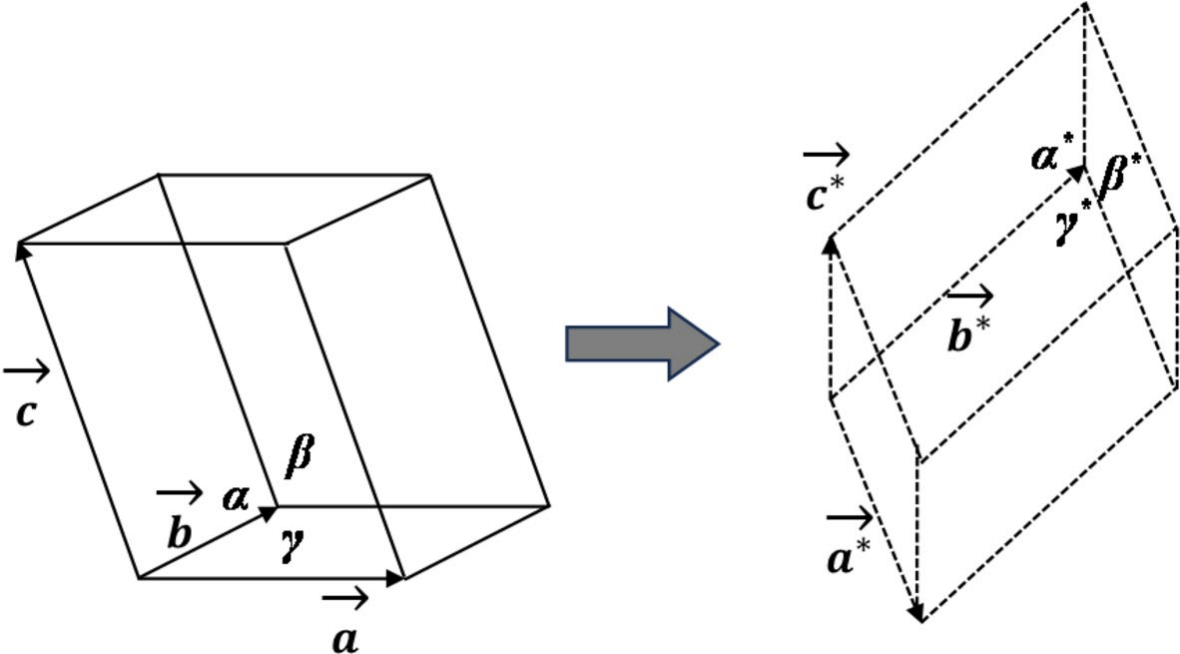
Schematic unit cells and basis vectors of a real-space crystal lattice and the corresponding reciprocal lattice.

As we know, in crystals, the atoms and molecules are arranged in a three-dimensional periodic pattern in real space, which leads to the formation of a crystal lattice. This real-space lattice corresponds to a reciprocal lattice in diffractive reciprocal space, composed of Bragg spots^11^. As illustrated in Fig. 4, the relationship between the real-space lattice and reciprocal lattice is defined by the basis vectors of both lattices, which is quantitatively presented in Eq. (1). Here, $\overrightarrow {{\varvec{a}}} ,\overrightarrow {{\varvec{b}}} ,\overrightarrow {{\varvec{c}}}$ are typically used to denote the basis vectors of the real space lattice, while $\overrightarrow {{{\varvec{a}}^{{\mathbf{*}}} }} ,\overrightarrow {{{\varvec{b}}^{{\mathbf{*}}} }} ,\overrightarrow {{{\varvec{c}}^{\user2{*}} }}$ are used to represent the basis vectors of the reciprocal lattice. This implies that if the lattice parameters and orientation of a crystal lattice are known, its diffraction pattern can be calculated. This is the fundamental physics for simulating 3D-RSM patterns.

1 $$\overrightarrow {{{\varvec{a}}^{{\mathbf{*}}} }} = \frac{{2\pi \overrightarrow {{\varvec{b}}} \times \overrightarrow {{\varvec{c}}} }}{{\overrightarrow {{\varvec{a}}} \cdot \left( {\overrightarrow {{\varvec{b}}} \times \overrightarrow {{\varvec{c}}} } \right)}},\overrightarrow {{{\varvec{b}}^{{\mathbf{*}}} }} = \frac{{2\pi \overrightarrow {{\varvec{c}}} \times \overrightarrow {{\varvec{a}}} }}{{\overrightarrow {{\varvec{b}}} \cdot \left( {\overrightarrow {{\varvec{c}}} \times \overrightarrow {{\varvec{a}}} } \right)}},\overrightarrow {{{\varvec{c}}^{\user2{*}} }} = \frac{{2\pi \overrightarrow {{\varvec{a}}} \times \overrightarrow {{\varvec{b}}} }}{{\overrightarrow {{\varvec{c}}} \cdot \left( {\overrightarrow {{\varvec{a}}} \times \overrightarrow {{\varvec{b}}} } \right)}}$$

In this study, to simulate the diffraction pattern accurately, we assume the substrate to be a (001)-oriented SrTiO_3_ with lattice parameters of *a* = *b* = *c* = 3.905 Å and *α* = *β* = *γ* = 90°. The epitaxial film is assumed to have various crystalline symmetries, lattice constants, and epitaxial relationships. For simplicity, the simulated diffraction spots are presented in the reciprocal lattice units of the SrTiO_3_ substrate, and the effects of lattice strain or lattice tilting due to lattice mismatch are not considered in this work.

The simulation process involves the following steps:

1. Assumption of Lattice Parameters: The substrate is assumed to be a perfect cubic crystal with known lattice parameters.
2. Epitaxial Film Characteristics: The epitaxial film is characterized by its crystalline symmetry, lattice constants, and the epitaxial relationship with the substrate.
3. Simulation of Diffraction Patterns: Using the known parameters, the diffraction patterns for films with different crystalline symmetries are simulated. The patterns are then analyzed to identify characteristic profiles that can facilitate the interpretation of experimental data.

By understanding the transformation between real space and reciprocal space, researchers can predict how changes in the crystal structure will manifest in the diffraction patterns.

### Lattice parameters calculation

The calculation method for lattice parameters involves several steps: Firstly, identify the diffraction spots originating from the same domain variant. Next, add the diffraction index. Then, Q coordinates of these diffraction spots are measured and expressed in linear equations as vector sum form of the reciprocal lattice basis vectors. More, the reciprocal lattice basis vectors are obtained by resolving these equations. Furthermore, the real space lattice basis vectors are calculated, according to the conversion formula between the reciprocal- and real-space basis vectors. Finally, the real-space lattice parameters and lattice orientation are obtained. By applying this calculation method to obtain the lattice parameters of the substrate and comparing them with the parameters on the Powder Diffraction File, the measurement error of this method can be evaluated.

## Data Availability

All data relevant to this study are contained within this published article.

## References

[1] Morita, T. & Cho, A. A hydrothermally deposited epitaxial PbTiO_3_ thin film on SrRuO_3_ bottom electrode for the ferroelectric ultra-High density storage medium. *Integr. Ferroelectr.*10.1080/10584580490894645 (2004).

[2] Fert, A. Origin, development, and future of spintronics (Nobel Lecture). *Angew. Chem. Int. Ed.*10.1103/RevModPhys.80.1517 (2008). 10.1002/anie.200801093 18626879

[3] Morita, T. & Cho, Y. Epitaxial PbTiO_3_ thin films on SrTiO_3_(100) and SrRuO_3_/SrTiO_3_(100) substrates deposited by a hydrothermal method. *Jpn. J. Appl. Phys.*10.1143/JJAP.43.6535 (2004).

[4] Son, J. Y., Bang, S. H. & Cho, J. H. Kelvin probe force microscopy study of SrBi_2_Ta_2_O_9_ and PbZr_0.53_Ti_0.47_O_3_ thin films for high-density nonvolatile storage devices. *Appl. Phys. Lett.*10.1063/1.1576916 (2003).

[5] Combs, N. G. et al. Ferroelectricity and superconductivity in strained Eu_x_Sr_1__−__x_TiO_3_ films. *Phys. Rev. B* **2**, 2. 10.1103/PhysRevB.107.094504 (2023).

[6] Van Weerdenburg, W. M. et al. Extreme enhancement of superconductivity in epitaxial aluminum near the monolayer limit. *Sci. Adv.*10.1126/sciadv.adf5500 (2023). 36857452 10.1126/sciadv.adf5500 PMC9977180

[7] Liu, W. L. & Wang, H. Flexible oxide epitaxial thin films for wearable electronics: Fabrication, physical properties, and applications. *J. Materiomics*10.1016/j.jmat.2019.12.006 (2020).

[8] Liu, W. L. et al. Mechanical strain-tunable microwave magnetism in flexible CuFe_2_O_4_ epitaxial thin film for wearable sensors. *Adv. Funct. Mater.*10.1002/adfm.201705928 (2018).32256277

[9] Liu, Y. X. et al. Van der Waals epitaxy for high-quality flexible VO_2_ film on mica substrate. *J. Appl. Phys.*10.1063/5.0046827 (2021).

[10] Xu, H. et al. Shear strain-induced anisotropic domain evolution in mixed-phase BiFeO_3_ epitaxial films. *AIP Adv.*10.1063/1.5080709 (2019).

[11] Xu, S. S. (ed.) *Advance in X-ray Diffraction* (Science Press, 1986).

[12] Zhao, Y. et al. Ultraflexible and malleable Fe/BaTiO_3_ multiferroic heterostructures for functional devices. *Adv. Func. Mater.*10.1002/adfm.202009376 (2021).

[13] Chen, X. Y. et al. Fabrication and structure analysis of freestanding BaTiO_3_–CeO_2_ epitaxial nanocomposite membranes. *Appl. Phys. Lett.*10.1063/5.0176672 (2023).

[14] Luo, Z. L. et al. Probing the domain structure of BiFeO_3_ epitaxial films with three-dimensional reciprocal space mapping. *Appl. Phys. Lett.*10.1063/1.4875579 (2014).24803681

[15] Xu, H. et al. Mixture domain states in PbTiO_3_ film with potentials for functional application. *Appl. Phys. Lett.*10.1063/1.5093798 (2019).

[16] May, S. J. et al. Quantifying octahedral rotations in strained perovskite oxide films. *Phys. Rev.*10.1103/PhysRevB.82.014110 (2010).

[17] Damodara, A. R. et al. Phase coexistence and electric-field control of toroidal order in oxide superlattices. *Nat. Mater.*10.1038/NMAT4951 (2017). 10.1038/nmat4951 28783161

[18] Gaudet, S. et al. Three dimensional reciprocal space measurement by x-ray diffraction using linear and area detectors: Applications to texture and defects determination in oriented thin films and nanoprecipitates. *J. Vac. Sci. Technol. A Vac. Surf. Films* **10**(1116/1), 4789984 (2013).

[19] Stoica, V. A. et al. Optical creation of a supercrystal with three-dimensional nanoscale periodicity. *Nat. Mater.*10.1038/s41563-019-0311-x (2019). 30886403 10.1038/s41563-019-0311-x

[20] An, F. et al. Highly flexible and twistable freestanding single crystalline magnetite film with robust magnetism. *Adv. Funct. Mater.*10.1002/adfm.202003495 (2020).34737689

[21] Cheema, S. S., Kwon, D., Shanker, N., Reis, R. D. & Salahuddin, S. Enhanced ferroelectricity in ultrathin films grown directly on silicon. *Nature*10.1038/s41586-020-2208-x (2020). 32322080 10.1038/s41586-020-2208-x

[22] Dong, G. H., Hu, Y., Guo, C. Q. & Liu, M. Self-assembled epitaxial ferroelectric oxide nano-spring with super-scalability. *Adv. Mater.*10.1002/adma.202108419 (2021). 35092066 10.1002/adma.202108419

[23] Li, C. J., Zou, M. J., Zhang, L., Wang, Y. & Wang, S. High-resolution X-ray diffraction analysis of epitaxial films. *Acta Metall. Sin.*10.11900/0412.1961.2019.00006 (2019).

[24] Liu, C. H. et al. Low voltage-driven high-performance thermal switching in antiferroelectric PbZrO_3_ thin films. *Science*10.1126/science.adj9669 (2023). 38096375 10.1126/science.adj9669

[25] Tan, C. B. et al. Engineering polar vortex from topologically trivial domain architecture. *Nat. Commun.*10.1038/s41467-021-24922-y (2021). 34330915 10.1038/s41467-021-24922-y PMC8324780

[26] Yang, Q. Q. et al. Ferroelectricity in layered bismuth oxide down to 1 nanometer. *Science*10.1126/science.abm5134 (2023). 36952424 10.1126/science.abm5134

[27] Zhang, J. F. et al. Super-tetragonal Sr_4_Al_2_O_7_ as a sacrificial layer for high-integrity freestanding oxide membranes. *Science*10.1126/science.adi6620 (2024). 38271502 10.1126/science.adi6620

[28] Zhang, Y. L. et al. Strain-driven Dzyaloshinskii–Moriya interaction for room-temperature magnetic skyrmions. *Phys. Rev. Lett.*10.1103/PhysRevLett.127.117204 (2021). 34558947 10.1103/PhysRevLett.127.117204

[29] Wang, R. X. et al. Phase coexistence and domain configuration in Pb(Mg_1/3_Nb_2/3_)O_3_–0.34PbTiO_3_ single crystal revealed bysynchrotron-based X-ray diffractive three-dimensional reciprocal space mapping and piezoresponse force microscopy. *Appl. Phys. Lett.*10.1063/1.4946776 (2016).27493276

[30] Saito, K. et al. Structural characterization of BiFeO_3_ thin films by reciprocal space mapping. *Jpn. J. Appl. Phys.*10.1143/JJAP.45.7311 (2006).

[31] Luo, Z. et al. Periodic elastic nanodomains in ultrathin tetragonal-like BiFeO_3_ films. *Phys. Rev. B*10.1103/PhysRevB.88.064103 (2013).

